# Fetal therapies and trials for lysosomal storage diseases: a survey of attitudes of parents and patients

**DOI:** 10.1186/s13023-022-02178-z

**Published:** 2022-01-29

**Authors:** Marisa E. Schwab, Julia E. H. Brown, Billie Lianoglou, Chengshi Jin, Patricia C. Conroy, Renata C. Gallagher, Paul Harmatz, Tippi C. MacKenzie

**Affiliations:** 1 Fetal Treatment Center, University of California, San Francisco, CA USA; 2 Center for Maternal-Fetal Precision Medicine, University of California, San Francisco, CA USA; 3 Department of Surgery, Division of Pediatric Surgery, University of California, 505 Parnassus Avenue, San Francisco, CA 94143 USA; 4 Program in Bioethics, University of California, San Francisco, CA USA; 5 Institute for Health & Aging, University of California, San Francisco, CA USA; 6 Biostatistics Core, Department of Surgery, University of California, San Francisco, CA USA; 7 Department of Pediatrics, University of California, San Francisco, CA USA

**Keywords:** Lysosomal storage disease, Mucopolysaccharidosis, Fetal therapy, Enzyme replacement therapy, Gene therapy, Patient attitudes, Clinical trial, ELSI (ethical, legal and social implications)

## Abstract

**Background:**

Lysosomal storage diseases (LSDs) are inherited metabolic disorders that may lead to severe multi-organ disease. Current ERTs are limited by anti-drug antibodies, the blood–brain barrier, and early disease onset and progression before ERT is started. We have opened a phase I clinical trial of enzyme replacement therapy (ERT) for fetuses with LSDs (NCT04532047). We evaluated the attitudes of parents and patients with LSDs towards fetal clinical trials and therapies.

**Methods:**

A multidisciplinary team designed a survey which was distributed by five international patient advocacy groups. We collected patients’ demographic, diagnostic, and treatment information. Associations between respondent characteristics and attitudes towards fetal therapies/trials were analyzed using multivariate ordinal logistic regression.

**Results:**

The survey was completed by 181 adults from 19 countries. The majority of respondents were mothers from the United States. The most common diseases were MPS1 (26%), MPS3 (19%), and infantile-onset Pompe (14%). Most patients (88%) were diagnosed after birth, at a median of 21 months. Altogether, 65% of participating patients and children of participants had received ERT, 27% a stem cell transplant, and 4% gene therapy. We found that half (49%) of respondents were unlikely to terminate a future affected pregnancy, 55% would enroll in a phase I clinical trial for fetal ERT, and 46% would enroll in a fetal gene therapy trial. Respondents who received postnatal ERT were significantly more likely enroll in a trial for fetal ERT or gene therapy (ERT OR 4.48, 95% CI 2.13–9.44, p < 0.0001; gene therapy OR 3.03, 95% CI 1.43–6.43, p = 0.0038). Respondents who used clinicaltrials.gov as a main source of information were more likely to choose to participate in a fetal trial (ERT OR 2.43, 95% CI 1.18–5.01, p = 0.016; gene therapy OR 2.86, 95% CI 1.27–6.46, p = 0.011).

**Conclusions:**

Familiarity with postnatal ERT increased respondents’ likelihood of pursuing fetal therapies. Families who use clinicaltrials.gov may be more receptive to innovative fetal treatments. The patient community has a favorable attitude towards fetal therapy; over half of respondents would enroll in a phase I clinical trial to assess the safety and efficacy of fetal ERT.

## Background

Lysosomal storage diseases (LSDs) are a group of heritable genetic diseases characterized by an enzyme deficiency that can lead to progressive, severe multi-organ disease. LSDs occur in 1 in 5,000 live births and are typically detectable in infancy, although prenatal diagnosis is also possible [1]. Enzyme replacement therapies (ERT) are effective therapies for children with various types of LSDs, including mucopolysaccharide diseases (MPS), Pompe disease, and Gaucher disease [2–7].

Although the availability of ERT has improved survival and quality of life for patients with numerous LSDs, this treatment can be limited due to several factors. First, ERTs cannot reverse disease sequelae that are already present at the time of treatment [8]. Importantly, some patients with LSDs develop complications before birth, such as the onset of hydrops fetalis [9]. Second, ERT given postnatally is not able to cross the blood brain barrier, with hematopoietic stem cell transplantation (HSCT) for MPS I as the only approved/standard of care therapy for these LSDs [10].Third, patients treated with ERT can develop anti-enzyme antibodies because their immune system recognizes the recombinant protein as a foreign antigen [11]. Some patients require immunomodulation to tolerate the ERT [12], and severe infusion reactions have been observed [4].

These limitations could potentially be addressed by in utero administration of ERT. We recently demonstrated that in utero ERT in a mouse model of MPS 7 improved survival of affected mice to birth [13]. Additionally, in utero ERT penetrated brain microglia and led to decreased microglial inflammation and improved neurological testing, such as grip strength, compared to mice only treated postnatally. Most importantly, in utero ERT prevented the development of anti-enzyme antibodies [13], consistent with prior reports of inducing immune tolerance after in utero administration of an antigen [14–17]. Based on these results and the severe unmet medical need in this group of patients, we have obtained an Investigational New Drug approval from the United States Food and Drug Administration to perform in utero ERT for fetuses with various LSDs for which an intravenous ERT is available (NCT04532047), including MPS 1, 2, 4a, 6, 7, neuronopathic Gaucher, Infantile-onset Pompe disease, and Wolman disease. In this trial, ERT is infused through the umbilical vein of the fetus every 2–4 weeks, a technique that is commonly used to administer blood transfusion for fetal anemia. If efficacious, in utero ERT may prevent not only disease progression in utero but may also mitigate some risks of postnatal ERT.

As preclinical studies are translated to human clinical trials, it is critical to consider their associated ethical, legal and social implications (ELSI) [18]. There has been increasing recognition of the importance of patient community involvement in clinical trial research [19, 20]. Patient organizations, and particularly those focused on rare diseases, have contributed to clinical trial design and recruitment of trial participants. Studies assessing the experiences of parents and patients with LSDs, such as evaluating health-related quality of life [21], perceptions of ERT [22], and newborn screening for LSDs [23], have demonstrated that parents and patients with LSDs are amenable to engaging with the research community. We thus partnered with patient organizations that represent the LSDs included in our phase I clinical trial to incorporate parent and patient perspectives as we consulted with regulatory authorities and developed the clinical trial protocol for in utero ERT.

In this study, we aimed to assess the attitudes of an international cohort of patients and parents of patients with certain LSDs regarding fetal ERT. Since many groups are also performing preclinical studies of in utero gene therapy for LSDs [24, 25], we also included questions on attitudes toward in utero gene therapy. We discuss our survey findings within the context of our phase 1 clinical trial for fetuses with certain LSDs and within the broader ELSI, including challenges around the extent to which in utero ERTs can offer socially inclusive treatment options in the context of the different healthcare systems around the world.

## Results

### Respondent characteristics and attitudes

A survey designed by a multidisciplinary team was distributed by five different patient advocacy groups (Table 1).

**Table 1:** Distribution of respondents (n = 181) by patient advocacy groups

Patient group	N (%)
National Mucopolysaccharidosis Society (USA)	100 (55.2%)
United Kingdom Mucopolysaccharidosis Society	36 (19.9%)
Duke Pompe Clinical and Research Group (USA)	24 (13.3%)
International Gaucher Alliance	12 (6.6%)
Instituto Vidas Raras (Brazil)	9 (5%)

The survey was completed by 181 adults from 19 countries. The majority of respondents (89%) were parents of patients diagnosed with a LSD, with a median age of 39 years (range 17–74) (Table 2). The most common diseases that patients and participants’ children had been diagnosed with were mucopolysaccharidosis (MPS) types 1 (26%) and 3 (18.8%) and Infantile-onset Pompe disease (13.8%). Over half of respondents (54.6%) had completed some type of college degree, with 16% having completed graduate level education.

**Table 2:** Characteristics of all survey respondents, including patients and parents of patients (n = 181)

Characteristic	n (%) or median (range)
Parent of affected patient	161 (89%)
Age (years)	39 (17–74)
Sex, female	159 (87.8%)
*Disease*	
Gaucher type 1	7 (3.9%)
Gaucher type 3	4 (2.2%)
Infantile-onset Pompe disease	25 (13.8%)
MPS 1	47 (26%)
MPS 2	23 (12.7%)
MPS 3	34 (18.8%)
MPS 4	21 (11.6%)
MPS 6	13 (7.2%)
MPS 7	2 (1.1%)
Other/Unknown MPS type	5 (2.8%)
*Highest level of education*	
Middle school/no formal schooling	5 (2.8%)
High school or diploma	28 (15.5%)
Some college	35 (19.3%)
Bachelor’s degree	35 (19.3%)
Graduate studies	29 (16%)
Unknown	49 (27.1%)

*MPS* Mucopolysaccharidosis

Most (87.8%) patients were diagnosed after birth, at a median of 21 months (range birth—51 years), Table 3. The median current patient age was 10 years (range birth—58 years). Most patients (78%) self-identified as white. Altogether, 65.2% of patients had received postnatal ERT, 27.1% a postnatal stem cell transplant, and 3.9% postnatal gene therapy.

**Table 3:** Characteristics of patients (including patient respondents and children of parent respondents) (n = 181)

Characteristic	n (%) or median (range)
*Diagnosed after birth*	159 (87.8%)
0–6 months	35 (19.3%)
7–36 months	72 (39.8%)
37–60 months	25 (13.8%)
> 60 months	26 (14.4%)
Unknown age	23 (12.7%)
Current age (years)	10 (0–58)
*Race/ethnicity*	
White	141 (77.9%)
Multiracial	18 (9.9%)
Asian	11 (6.1%)
Other/unknown	6 (3.4%)
Black	2 (1.1%)
Hispanic or Latino	2 (1.1%)
American Indian or Alaska Native	1 (0.6%)
Received postnatal ERT	118 (65.2%)
Received stem cell transplant	49 (27.1%)
Received gene therapy	7 (3.9%)
Clinicaltrials.gov is their main source of information	44 (24.3%)
*Type of healthcare coverage*	
Employer-based	72 (39.8%)
Public (including national healthcare systems)	87 (48.1%)
Privately purchased by a parent	12 (6.6%)
No coverage	4 (2.2%)
Other/Unknown	6 (3.3%)

*ERT* enzyme replacement therapy

**Fig. 1:**
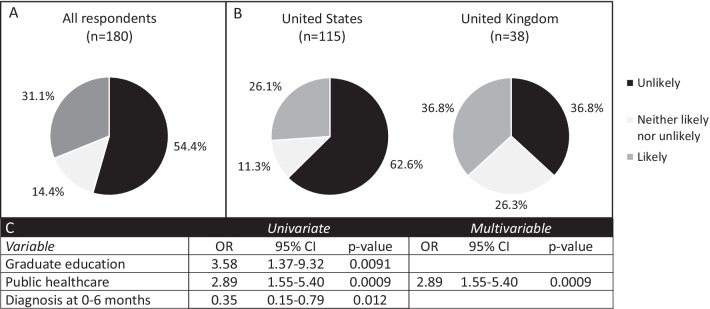
Attitudes toward continuation of a future pregnancy affected with an LSD. Respondents were asked the question “If you or your partner were to become pregnant and the fetus were diagnosed with MPS, how likely would you be to end the pregnancy?” **A** All respondents (n = 180). **B** Comparison of respondents from the United States (n = 115) and the United Kingdom (n = 38) (p = 0.012, Fisher’s exact test), **C** Variables with statistical significance on univariate and multivariable regression. *OR* odds ratio, *CI* confidence interval

Pregnancy choices after a diagnosis of a severe genetic condition in the fetus are personal and complex. Since LSDs are not screened for in every pregnancy, we surmised that most patients would be diagnosed due to known carrier status in the parents, often due to the diagnosis of a previous child with an LSD. We therefore asked parents about their likelihood of ending a future affected pregnancy. For international patients, we only analyzed responses from countries in which pregnancy termination is legal. Over half (54.4%) of respondents said they were unlikely to terminate an affected pregnancy, while 31.1% were likely to do so (Fig. 1). When comparing parent and patient responses, 32% of parents and 19% of patients were likely to end a future affected pregnancy (p = 0.4). Since the majority of respondents were from the United States (n = 115) or the United Kingdom (n = 38), we also compared the responses between these groups. Respondents from the United States were significantly less likely to terminate a future affected pregnancy (26.1% vs 36.8%, p = 0.012). On multivariable regression, respondents were significantly more likely not to continue a future affected pregnancy if they had some form of public healthcare insurance (including the British National Health Service and United States Medicare/Medicaid) (OR 2.89, 95% CI 1.55–5.40, p = 0.0009).

**Fig. 2:**
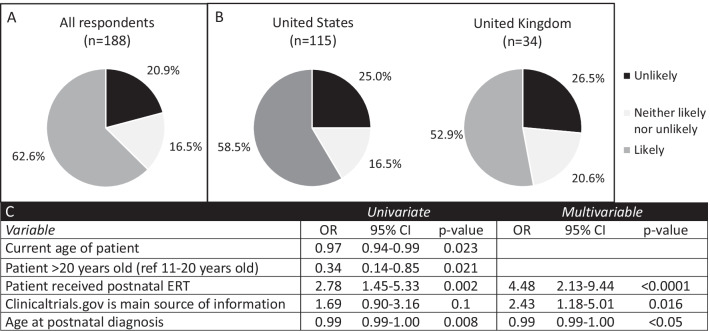
Attitudes toward enrolling in a phase 1 clinical trial for fetal enzyme replacement therapy. Respondents were asked the question “If you or your partner were to become pregnant and the fetus were diagnosed with MPS, how likely would you be to enroll in a phase I clinical trial (to determine safety) for fetal enzyme replacement therapy?” **A** all respondents (n = 188), **B** Comparison of respondents from the United States (n = 115) and the United Kingdom (n = 34) (p = 0.58, Fisher’s exact test), **C** Variables with statistical significance on univariate and multivariable regression. *Or* odds ratio, *CI* confidence interval, *ERT* enzyme replacement therapy

As we developed our clinical trial for fetal enzyme replacement therapy, we sought to assess the community’s opinion of a phase I trial. With respect to participation in a phase 1 clinical of ERT, 62.6% were likely to enroll in a phase I clinical trial for fetal ERT while 20.9% were unlikely (Fig. 2). When comparing the responses of patients versus parents, 65% of patients would self-enroll and 60% of parents would enroll their affected child (p = 0.8). There was no significant difference between respondents from the United States versus those from the United Kingdom (58.5% vs 52.9%, p = 0.58). On univariate regression, patients older than 20 years old at the time of survey completion (and their parents) were less likely to want to enroll than younger patients. On multivariable regression, respondents were significantly more likely to enroll in a trial for fetal ERT if their child had received postnatal ERT (OR 4.48, 95% CI 2.13–9.44, p < 0.0001), and if they used clinicaltrials.gov as their main source of information (OR 2.43, 95% CI 1.18–5.01, p = 0.016). Having a child who received stem cell transplantation did not appear to affect parents’ attitudes regarding fetal therapy. When comparing the 165 respondents from countries in which pregnancy termination is allowed after the first trimester (Australia, England, Greece, India, Netherlands, Norway, Scotland, Serbia, South Africa, Sweden, United States, Wales) and the 16 respondents from countries in which termination is illegal (Argentina, Brazil, Guatemala, Northern Ireland, Peru, Slovenia), there was no difference in their likelihood of participating in a trial for fetal ERT (61% vs 55%, p = 0.75).

**Fig. 3:**
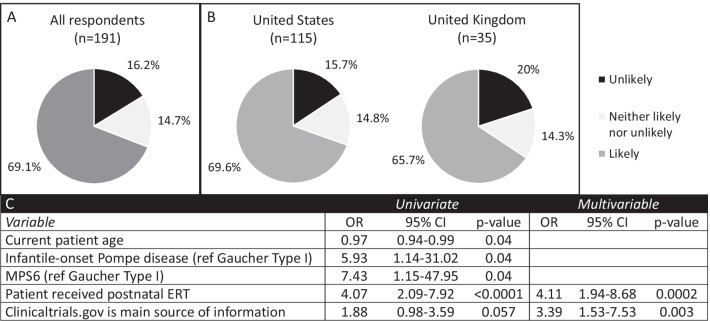
Attitudes toward choosing an approved fetal enzyme replacement therapy. Respondents were asked the question “If you or your partner were to become pregnant and the fetus were diagnosed with MPS, choose fetal enzyme replacement therapy?” **A** All respondents (n = 191), **B** Comparison of respondents from the United States (n = 115) and the United Kingdom (n = 35) (p = 0.8, Fisher’s exact test), **C** Variables with statistical significance on univariate and multivariable regression. *MPS6* Mucopolysaccharidosis type 6, *OR* odds ratio, *CI* confidence interval, *ERT* enzyme replacement therapy

If clinical trials of fetal ERT show a benefit, it is possible that this treatment could become an approved therapy for routine clinical care, rather than a research protocol. We therefore asked whether respondents would be more likely to consider this approach as an approved therapy and found that 69.1% would be likely to choose fetal ERT if it was an approved therapy compared to 16.2% who reported they were unlikely to do so (Fig. 3). When we compared those who were likely to choose an approved fetal ERT versus those likely to enroll in a phase I trial for fetal ERT, it trended towards significance (p = 0.0521). Overall, most parents and patients (69% and 67%, respectively) would choose an approved fetal ERT (p > 0.99 when comparing parents versus patients). There was no significant difference between respondents from the United States and the United Kingdom (69.6% vs 65.7%, p = 0.8). Respondents were significantly more likely to choose an approved fetal ERT for a future affected pregnancy if their child had received postnatal ERT (OR 4.11, 95% CI 1.94–8.68, p = 0.0002) or if they used clinicaltrials.gov as a main source of information about their disease (OR 3.39, 95% CI 1.53–7.53, p = 0.003).

**Fig. 4:**
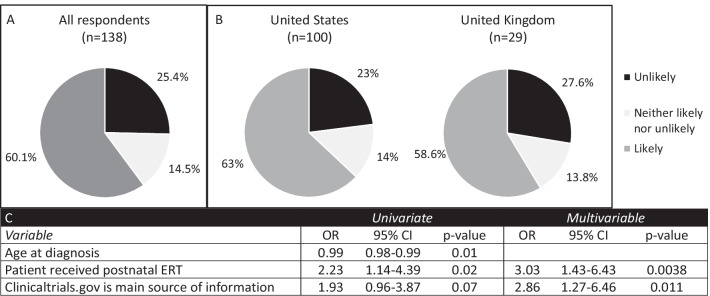
Attitudes toward participation in a clinical trial for fetal gene therapy. Respondents were asked the question “If you or your partner were to become pregnant and the fetus were diagnosed with MPS, enroll in a phase I clinical trial (to determine safety) for fetal gene therapy?” **A** All respondents (n = 138), **B** Comparison of respondents from the United States (n = 100) and the United Kingdom (n = 29) (p = 0.87, Fisher’s exact test), **C** Variables with statistical significance on univariate and multivariable regression. *OR* odds ratio, *CI* confidence interval, *ERT* enzyme replacement therapy

Given the potential availability of clinical trials of in utero gene therapy in the future, we also included a question regarding the likelihood of enrolling in a phase I clinical trial for fetal gene therapy. We found that 60.1% of respondents were likely to enroll while 25.4% were unlikely to do so (Fig. 4). Both parents (60%) and patients (60%) reported being likely to participate in a trial for fetal gene therapy (p > 0.99 when comparing parents versus patients). There was no significant difference between respondents from the United States versus those from the United Kingdom (63% vs 58.6%, p = 0.87). Respondents were significantly more likely to enroll in a fetal gene therapy phase I trial if the patient had received postnatal ERT (OR 3.03, 95% CI 1.43–6.43, p = 0.0038) and if they used clinicaltrials.gov (OR 2.86, 95% CI 1.27–6.46, p = 0.011).

## Discussion

In this international survey of patients and parents of patients with certain types of LSDs, we found that the patient/parent community has a favorable attitude towards fetal therapies. Most families would opt to continue a future affected pregnancy, and most would consider enrolling in a research study to assess the safety and efficacy of fetal ERT. The responses from American and British patients/parents were largely consistent. Familiarity with ERT (that is, having a child who had received postnatal ERT) was associated with a more favorable attitude towards choosing fetal ERT and enrolling in a phase I clinical trial for fetal ERT. Respondents were also more likely to enroll in any fetal therapy if they obtained their information from clinicaltrials.gov, which may be a proxy for their facility with searching and understanding novel clinical research protocols. On univariate regression, having a younger child at the time of survey completion was associated with a higher likelihood of enrolling in a phase I trial for fetal enzyme replacement therapy. This may be a proxy for disease severity, wherein parents of children with more a severe LSD may be more likely to pursue new therapies and enroll in clinical trials.

Our results are similar to those of the European EVERREST Prospective Study, which investigated the attitudes toward a phase I clinical trial of in utero Vascular Endothelial Growth Factor (VEGF) to treat severe early-onset fetal growth restriction (FGR) among women who had previously enrolled in the EVERREST Study [26]. The authors highlighted the willingness of respondents to participate in research and found that mothers’ experience with FGR shaped enthusiasm for future clinical trials. In our study, familiarity with postnatal ERT, including its significant benefits, may have led patients and parents to consider choosing ERT during the prenatal period.

The demographics and preferences of survey respondents for prenatal ERT research and clinical trials point to broader ELSI. Although there was not a significant difference between respondents from the United States and the United Kingdom regarding the likelihood of choosing an approved fetal ERT (69.6% vs 65.7%), there was a larger difference when it came to enrolling in a clinical trial for fetal ERT (62.6% in the United States vs 52.9% in the United Kingdom), which may be explained by differences in healthcare coverage. In the absence of universal healthcare in the United States, clinical trials become an avenue for more affordable treatment. Predictably, access to public healthcare shaped survey respondents’ preferences to terminate future affected pregnancies. Most survey respondents were unlikely to terminate future affected pregnancies. However, respondents with access to public healthcare were more likely to terminate. Respondents from the United Kingdom were more likely to terminate compared to those from the United States (36.8% vs 26.1%).

Patient and parent preferences are shaped by access to treatments, which are highly contingent on socioeconomic, political, and racial factors. Given that 78% of respondents identified as white and 55% were, at minimum, college-educated, our findings should be interpreted in the context of larger health inequity concerns. Individuals who join patient associations may be more likely to have a higher educational level; this may be a source of bias in our study. Worldwide experience with all LSD types has uncovered no known founder effect or specific geographic predilection [27–29]. It is important to consider the international translation of clinical trials for novel drug treatments in resource-poor countries along with the development of collaborative, international data sets [30]. Disparities within countries should also be considered. Many of the ERT clinical trials in patients with MPS enroll predominantly white patients [31, 32]. Future inquiries must ask how under-represented groups might participate in these fetal therapies and trials. In certain countries, receiving a prenatal diagnosis is not a ‘choice’ if there are external structural limitations that render some options impracticable [33]. Although we did not ask survey respondents about their belief systems, this is another variable that should be accounted for when considering what options prospective patients may feel they have [34]. Preferences for experimental fetal therapies may also be partly explained by the older age category of parents in our survey (median age of 39), which highlights the need to ensure that clinical trial design is sensitive to parents at different life stages, and that benefit-to-risks are weighed accordingly.

There are limitations to this study. We cannot determine if the reported favorable attitudes would translate to actual treatment and trial participation choices. There may have been a respondent bias; those who are members of LSD patient organizations and chose to participate may be more invested in research aimed at developing future therapies such as fetal treatment. Our survey was conducted during the COVID-19 pandemic, which may have exacerbated barriers of participation for under-represented groups. The timing of being approached and familiarity with clinical trials and the research team may also be critical to minimize the stress burden while enrolling in a trial during pregnancy [26]. Furthermore, our findings should be interpreted within clinical demographic contexts. First, we did not analyze respondent attitudes separately for each disease type, due to the variability in available therapies among patients and over time. Since the first ERT was approved for MPS I in 2003 [35], the landscape of ERT for LSDs has continued to dramatically change over the past two decades. Our survey did not differentiate between patients who had not received a therapy due to lack of access versus personal preference. As the standard of care for patients with different LSDs continues to rapidly evolve, classifying patients based on their disease type may not accurately reflect the multiple avenues available to patients at any given time. Additionally, the correlation between enthusiasm for prenatal ERT and prior experience with postnatal ERT and use of clinical.trials.gov points to established trustworthiness in innovative healthcare and research, which is not generalizable to all members of a patient community.

The strengths of the study are that we were able to reach a large number of respondents affected by certain LSDs in multiple countries. We were therefore able to obtain insights into potential connections for patient preferences, such as the availability of public health insurance. Although qualitative research assessing patients’ and families’ attitudes towards genetic diseases and postnatal treatment options for conditions such as spinal muscular atrophy (SMA) and LSDs has been performed [36–42], there has been limited research into attitudes towards emerging fetal therapies. Partnering with robust national patient advocacy groups can rapidly yield in-depth, reliable insights into community stakeholder attitudes towards novel therapies. Future qualitative work exploring the underlying reasons behind respondents’ answers to our survey through, for instance, semi-structured interviews or focus groups, will allow for a more comprehensive discussion about prenatal diagnoses, preimplantation genetic diagnosis, pregnancy choices and related decision-making. Given the vast social inclusion barriers, wide stakeholder engagement is critical to comprehensively redress concerns as genomic medicine continues to evolve [43].

## Conclusions

This is the first stakeholder survey conducted for fetal therapies for patients with certain LSDs and offers meaningful insights into patient and parent attitudes towards emerging fetal therapies. The international LSD community surveyed expressed a favorable attitude towards in utero enzyme replacement therapies for LSDs, including enrollment in future clinical trials. Although we identified several racial and socioeconomic biases in our sample that point to social inclusion barriers, this study serves as an important starting point for further inquiry into direct stakeholder views as well as ELSI. Ongoing patient and parent engagement will be critical as we enroll patients with certain LSDs in a phase I clinical trial of in utero ERT.

## Methods

### Instrument development

The aim of this study was to assess the attitudes of patients and parents of patients with certain LSDs towards fetal treatment and fetal clinical trials. No validated questionnaire was available on this topic; thus, we devised an electronic questionnaire that was reviewed by a multidisciplinary group (genetic counselor, sociologist, fetal surgeon, patient advocacy leaders) (Appendix 1) to determine face and construct validity. The survey items were modified according to feedback in multiple iterations. Questions were written in a structured response format with a free text option where appropriate. The survey contained 20 questions. After determining whether the respondent was a patient or a parent of a patient, the survey was divided into three sections. The first enquired about diagnostic details and therapies received by the affected individual (if the respondent was a patient, themselves; if the respondent was a parent, their child or children). The second section evaluated attitudes towards potential fetal therapies and trials using a 5-point Likert scale. The third section appraised respondents’ demographic characteristics, education level, and healthcare coverage.

### Survey distribution

The survey was published on the survey platform, Qualtrics XM (SAP, Provo, UT). A link to the survey was posted on the social media pages and in the monthly newsletters of various patient advocacy groups: National MPS Society (United States), the UK MPS Society (United Kingdom), Vidas Raras (Brazil), Gaucher Alliance (international), and the Duke Pompe Research Society (United States) between February 2020 and January 2021. A response rate could not be calculated given the fluctuating nature of social media, which was used for distribution. Potential participants were counseled that the survey was optional and completely anonymous. There were no benefits to participating, and respondents were not compensated. A cover letter explained the purpose of the survey, our published results of fetal ERT in a mouse model of MPS 7 [13], and the possible risks and benefits of fetal therapy for certain LSDs (Appendix 2). Informed consent was obtained prior to survey completion. Given the nature of the survey distribution, it was not possible to calculate a response rate.

### Statistical analysis

Survey responses with less than 80% of questions answered were excluded. Consequently, the denominator for each variable differed depending on the number of responses for each specific question. Descriptive statistics and ordinal logistic regression were used to analyze associations between respondent characteristics and their attitudes towards fetal therapy and trials. A two-sided alpha of 0.05 was considered significant for all analyses. Univariate ordinal regression incorporating all respondent and parent characteristics was initially performed. Subsequently, multivariate ordinal regression including any significant variables on univariate regression was performed. For the outcome variables measured using a 5-point Likert scale, we performed ordinal logistic regression both with all 5 possible outcomes and with 3 outcomes by combining “very unlikely”/ “somewhat likely”, and “very likely”/ “somewhat likely.” All statistical analyses were performed using SAS v. 9.4 (SAS Institute Inc, Cary, NC).

## Data Availability

The datasets used and/or analyzed during the current study are available from the corresponding author on reasonable request.

## Appendix 1: Survey

**Table:**

I am a patient with MPS	I am a parent of a patient with MPS
1) I have been diagnosed with MPS	1) My child or fetus was diagnosed with MPS
a. Type I (Hurler, Hurler-Scheie, or Scheie Syndrome)	a. Type I (Hurler, Hurler-Scheie, or Scheie Syndrome)
b. Type II (Hunter Syndrome)	b. Type II (Hunter Syndrome)
c. Type III (Sanfilippo Syndrome)	c. Type III (Sanfilippo Syndrome)
d. Type IV (Morquio Syndrome)	d. Type IV (Morquio Syndrome)
e. Type VI (Maroteaux-Lamy Syndrome)	e. Type VI (Maroteaux-Lamy Syndrome)
f. Type VII (Sly Syndrome)	f. Type VII (Sly Syndrome)
g. Type IX (Hyaluronidase deficiency)	g. Type IX (Hyaluronidase deficiency)
h. Other: (free text)	h. Other: (free text)

1. I have:
  - One child with MPS
  - Multiple children with MPS

**Section 1: Attitudes**

2) At what age was (were) your child(ren) diagnosed?
  - Before birth: gestational age:
  - After birth:
3) If you are a woman, how many pregnancies have you had with a diagnosis of MPS?
4) Where do you get information about treatment options?
  - Physicians
  - Patient organizations (such as the National MPS Society)
  - Other parents of MPS patients
  - Clinicaltrials.gov
  - Other, please specify:
5) My child(ren) has (have) received enzyme replacement therapy.
  - Yes
  - No
6) My child(ren) has (have) received a hematopoietic stem cell transplant.
  - Yes
  - No
7) My children have received gene therapy.
  - Yes
  - No
8) If you or your partner were to become pregnant and the fetus were diagnosed with MPS, how likely would you be to:
9) How old are you?
  **Table:**
  	Extremely unlikely	Somewhat unlikely	Neither likely nor unlikely	Somewhat likely	Extremely likely
  End the pregnancy?					
  Choose fetal enzyme replacement therapy?					
  Enroll in a phase I clinical trial (to determine safety) for fetal **enzyme replacement therapy**?					
  Enroll in a phase I clinical trial (to determine safety) for fetal **gene therapy**?					

**Section 2: Demographics**

10) What is your gender?
  - Female
  - Male
  - Other
11) How old is (are) your child(ren)?
12) What is your occupation?
13) What type of health coverage does (do) your affected child(ren) have?
  - Employer-based
  - Privately purchased by a parent
  - Medicaid
  - Other public
  - They do not currently have health coverage
  - Other:
14) What is the highest level of education you have received?
  - Middle school
  - High school diploma or GED
  - Some college
  - Bachelor’s degree
  - Graduate studies
  - No formal schooling
15) How many pregnancies have you had with a diagnosis of MPS?
16) What is (are) the geographic ancestry of your child(ren)? (select all that apply)
  - American Indian or Alaska Native
  - Asian
  - Black or African American
  - Native Hawaiian or Other Pacific Islander
  - White
  - Other, please specify:

## Appendix 2

**Cover letter** Currently, an FDA-approved ERT is used to treat children with MPS7 to slow the progress of the disease. In our lab, studies of mice with MPS7 yielded better outcomes when mice were treated with ERT before birth versus after birth.

**Possible benefits of fetal therapy**: ERT given prenatally may confer similar benefits to human fetuses affected by MPS7. Beyond MPS7, we think that patients with other MPS disorders would benefit from treatment before birth. For example, one of the main limitations of the current therapy is developing an allergic reaction to the enzyme: treating before birth could induce tolerance to the missing protein, which would reduce complications of ERT after birth. Treating the fetus also allows the enzyme to penetrate the blood brain barrier, possibly improving neurologic outcomes. Finally, some babies are already born with irreversible damage in organs such as the heart and the brain and fetal therapy could prevent these complications.

**Possible risks of fetal therapy**: Every treatment has risks and benefits. While fetal surgery and other therapies are routinely performed at multiple centers around the world, fetal enzyme therapy has not been given to a patient before. We anticipate that the risks of giving the enzyme (which is FDA approved for patients after birth) would be minimal. However, there may be a risk from the procedure of entering the umbilical vein of the fetus to give the injection: this is a treatment that is commonly performed for blood transfusion.

**How you can help**: We would like to understand patient and parent preferences on prenatal treatment of MPS. Information we collect that could identify you will be secured separately from responses. Input from patients and parents is an essential part of developing new therapies, and we are grateful for your participation.
